# No evidence for niche segregation in a North American Cattail (*Typha*) species complex

**DOI:** 10.1002/ece3.225

**Published:** 2012-05

**Authors:** Andrew McKenzie-Gopsill, Heather Kirk, Wendy Van Drunen, Joanna R Freeland, Marcel E Dorken

**Affiliations:** Department of Biology, Trent University1600 West Bank Drive, Peterborough, ON, K9J 7B8, Canada

**Keywords:** hybridization, invasive species, SSR markers, *Tyhpa × glauca*, water depth

## Abstract

Interspecific hybridization can lead to a breakdown of species boundaries, and is of particular concern in cases in which one of the parental species is invasive. Cattails (*Typha* spp.) have increased their abundance in the Great Lakes region of North America over the past 150 years. This increase in the distribution of cattails is associated with hybridization between broad-leaved (*Typha latifolia*) and narrow-leaved cattails (*T. angustifolia*). The resulting hybrids occur predominantly as F_1_s, which are known as *T. × glauca*, although later-generation hybrids have also been documented. It has been proposed that in sympatric populations, the parental species and hybrids are often spatially segregated according to growth in contrasting water depths, and that this should promote the maintenance of parental species. In this study, we tested the hypothesis that the two species and their hybrids segregate along a water-depth gradient at sites where they are sympatric. We identified the two parental species and their hybrids using molecular genetic markers (SSR), and measured shoot elevations (a proxy for water depth) at 18 sites in Southern Ontario, Canada. We found no evidence for niche segregation among species based on elevation. Our data indicate that all three lineages compete for similar habitat where they co-occur suggesting that there is potential for an overall loss of biodiversity in the species complex, particularly if the hybrid lineage is more vigorous compared to the parental species, as has been suggested by other authors.

## Introduction

Adaptation of congeneric species to different environmental conditions can play a key role in the maintenance of species barriers (Schluter 2009), particularly when congeners occur sympatrically (Seehausen et al. 2008). For example, niche segregation should promote reproductive isolation via the spatial isolation of otherwise interfertile species (reviewed by Sobel et al. 2010 in the context of ecological speciation). Alternatively, if reproductive isolation is incomplete and hybridization occurs, parents and hybrids may still become spatially segregated into contrasting microhabitats (e.g., Mao and Wang 2011), particularly in highly heterogeneous habitats and in cases where genotype-by-environment interactions regulate the fitness of each lineage (Arnold and Martin 2010). For example, *Ipomopsis aggregata* and, *I. tenuituba* are distributed at opposite ends of an elevational gradient with hybrids occurring at mid-elevation sites. In a reciprocal transplant experiment, each of the lineages performed best in the habitat in which it was from, suggesting the potential for lineage sorting and the maintenance of parental lineages at opposite ends of the cline (Campbell and Waser 2007).

The cattail species *Typha angustifolia* (narrowed-leaved cattail) and *T. latifolia* (broad-leaved cattail) frequently co-occur throughout their ranges in both North America and Europe (Galatowistch et al. 1999). In North America, *T. latifolia* is generally regarded to be a native species, but it is not yet clear whether *T. angustifolia* is native to North America (Shih and Finkelstein 2008), or introduced from Europe (Stuckey and Salamon 1987). Pollen and herbarium records from eastern North America show that despite a period of rapid range expansion for *T. angustifolia* in the early-to-mid 20th century, the ranges of *T. angustifolia* and *T. latifolia* have subsequently increased at the same rate (Shih and Finkelstein 2008), and the frequency of mixed stands of both species has also increased. Interspecific hybrids are found in many regions where the two species co-occur, and the resulting hybrid species (*T. × glauca*) has been implicated in a recent invasion of cattails in parts of north-eastern North America (Shih and Finkelstein 2008), particularly in regions around the Great Lakes (Frieswyk and Zedler 2007; Travis et al. 2010; Kirk et al. 2011). Until recently, it was widely thought that *T. × glauca* was a sterile hybrid that occurred only as an F_1_ (e.g., Kuehn et al. 1999) and spread clonally via the formation of rhizomes. However, recent studies have provided evidence for low-to-moderate levels of backcrossing and intercrossing in natural hybrid populations (Travis et al. 2010; Kirk et al. 2011; Travis et al. 2011).

Water depth is a key environmental determinant of macrophyte community structure, (Sculthorpe 1967; Lacoul and Freedman 2006), and also imposes a considerable selection pressure on aquatic plants (e.g., Colmer and Voesenek 2009). Accordingly, water depth is thought to play an important role in the maintenance of species barriers for aquatic plants (Anderson 1948; Johnston et al. 2001), including *Typha* (Grace and Wetzel 1981; Travis et al. 2010). Although *Typha* species often occur sympatrically, it has been argued that they partition habitats within sites with *T. latifolia* growing in shallower water than *T. angustifolia* (Grace and Wetzel 1981; Travis et al. 2010). Moreover, there is some evidence that *T. × glauca* favors low or fluctuating water levels (Smith 1967; Lishawa et al. 2010). In at least some sites, reproductive isolation between the species seems to be maintained by temporal separation in flowering times (Selbo and Snow 2004), though it is still not clear why there is regional variation in the frequency of hybridization events (see Selbo and Snow 2004; Kirk et al. 2011). The apparent subdivision of aquatic habitats along water-depth gradients by *T. latifolia* and *T. angustifolia* has led to the prediction that niche segregation should enable the maintenance of parental species within mixed stands (Travis et al. 2010). Specifically, Travis et al. (2010) hypothesized that *T. latifolia*'s preference for shallow water may provide the species with a refuge in mixed stands. Niche segregation of these lineages may therefore provide a mechanism for the maintenance of species diversity over the long term, and native *T. latifolia* in particular. In contrast, if no niche segregation occurs, and one lineage can competitively exclude the others in all habitats, there is greater potential for loss of biodiversity at the regional level (as per Seehausen et al. 2008). Indeed, hybrid cattails are often reported to be competitively superior to both parental species (reviewed in Galatowitsch et al. 1999), although we are not aware of any direct tests of this idea.

In this study, we examined evidence for niche segregation between cattails at 18 sites within the eastern Great Lakes region, an area in which both parental species and *T. × glauca* are often found in mixed stands, and the hybridization process appears to be ongoing (Kirk et al. 2011). Specifically, we tested the hypothesis that *T. latifolia*, *T. angustifolia*, and *T. × glauca* occupy contrasting water depths in sites where all three lineages co-occur. In order to estimate water depths (which can fluctuate seasonally), we measured shoot elevations within sites, and compared elevations of all three lineages across multiple sites.

## Materials and Methods

### Field surveys

#### Site criteria

**Figure 1:**
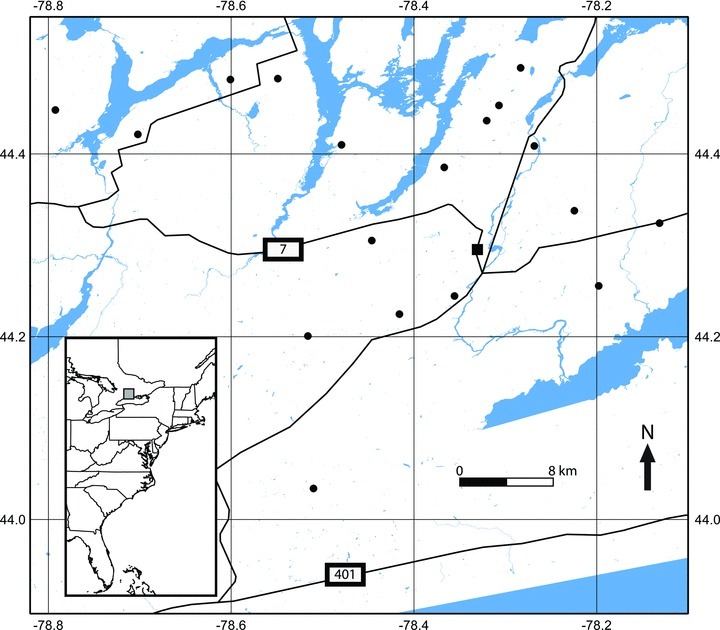
Map of the 18 sites included in this study. The black square in the center of the map indicates the location of the city of Peterborough. Numbers within boxes indicate the identity of major E-W highways in the region. The gray square in the inset shows the location of the study area. Blue shading indicates the location of water bodies.

Our primary goal was to evaluate differences in water depth between *T. latifolia* and co-occurring congeners using shoot elevations as a proxy for water depth. For the following two reasons, our sampling strategy emphasized the number of sites sampled over the number shoots sampled per site. First, the plants growing at any particular location share idiosyncratic colonization histories and edaphic conditions, reducing the statistical independence between shoots within samples. Second, because cattails are clonal, sampled shoots might often share identical genotypes, further reducing the independence of observations made at the shoot level. Accordingly, we sampled as many sites as could be found in the area surrounding Peterborough, Ontario, between late June and late August 2010 that met the following two criteria. First, sites had to have a mixture of two phenotypes that, using morphological criteria, including leaf width, the separation of female and male inflorescences segments, and plant versus inflorescence heights (Grace and Harrison 1986; Kuehn and White 1999), were likely to include *T. latifolia*, with the remaining phenotypes representing *T. angustifolia* or *T. × glauca*, but preferably both. Second, the site had to be large enough to enable the sampling of shoots along a transect through contiguous patches of the two (or three) phenotypes with at least 2 m between each sampled ramet, up to a maximum of 100 m. Our final sample included 265 ramets, sampled from 18 sites ranging from roadside ditches to large, relatively undisturbed wetlands. Twelve of the 18 sites included samples representing all three species (Fig. 1).

#### Elevations

The hypothesis for the maintenance of *T. latifolia* with its congeners tested here, and suggestions made in the literature about ecological differences between *T. latifolia* and its congeners, specifically mention water depth. Because water depths can vary seasonally within sites, we chose instead to measure shoot elevations (i.e., elevation measured from the base of the shoot) encountered along our transects. Indeed, because sites sometimes have no standing water towards the end of the summer (i.e., when the reproductive structures that enable tentative species assignments are present), this approach should have provided a more accurate estimate of average annual water depth than would one or a small number of water depth measurements at each site during the growing season. In particular, zero values of water depth in drained habitat provide no information about differences in water depths among shoots at that site when it is flooded. Water depths and elevations are often used as proxies for one another in studies by ecohydrologists and aquatic plant ecologists (e.g., Watts et al. 2010; Lawrence and Zedler 2011; also see Eppley 2001 and Johnston et al. 2001 for similar approaches). Elevation measurements were made using a Leica Disto D8 (Leica Geosystems, St. Gallen, Switzerland), a laser range finder that can estimate elevations at the millimeter scale by measuring the distance and angle between a fixed point and the sampled shoot. The Disto D8 was mounted on a tripod and elevations were measured by bouncing the laser off a leveling rod with a fixed, reflective target at its top that was placed at the base of each sampled shoot whether or not the base was submerged. Because shoots were sampled within contiguous habitat, differences in elevations among shoots within sites should directly correspond to differences in water depth (i.e., the height of the plant above/below the water line). In addition to shoot elevations, we also measured the shoot height (measured as the length of the longest leaf per shoot) for use as a covariate in the analyses described below; shoot height is known from previous studies to be positively correlated with water depth (e.g., Travis et al. 2010).

### Microsatellite genotyping

From each shoot, we removed a 7-cm leaf segment from the tip of the youngest leaf. The leaf sample was then placed in an airtight bag together with Sorbead orange silica beads (eCompressedair, Westfield, MA, U.S.A.). Leaf samples were dried at room temperature for two days and then stored at –20°C until DNA isolation could be performed. We extracted and isolated DNA from each leaf sample using the E.Z.N.A. Plant DNA Kit (Omega Bio-Tek Inc., Norcross GA, U.S.A.), according to the manufacturer's protocol for dry specimens. For each extraction, we used approximately 40 mg of powered leaf tissue. Each sample was eluted to a final volume of 100 µl. Plants were genotyped at each of six microsatellite loci developed by Tsyusko-Omeltchenko et al. (2003; TA3, TA5, TA7, TA8, TA16, TA20). For each locus, the forward or reverse primer was fluorescently labeled with either hex (TA3F, TA5F, TA15R), fam (TA8F, TA16R, TA20F), or ned (TA7F). Amplifications were performed as one multiplex and five singleplex reactions, each in a total volume of 10 µl according to Kirk et al. (2011). Genotyping was carried out on an ABI 3730 DNA Analyzer (Applied Biosystems Inc., Foster City, CA, U.S.A.). Fragments were sized using GeneMarker® software v.1.6 (SoftGenetics LLC., State College, PA, U.S.A.), with ROX 500 (Applied Biosystems Inc.) size standard for reference. Three samples did not amplify fully across all loci and were removed from the analyses described below.

### Species assignment

To assign species/hybrid status to each shoot, we used a diagnostic key (Kirk et al. 2011) that can distinguish between *T. latifolia* and *T. angustifolia* based on the presence of private alleles. Plants that contained all *T. latifolia* alleles were classed as pure *T. latifolia*, those that contained all *T. angustifolia* alleles were classed as pure *T. angustifolia* and individuals that shared one allele from *T. latifolia* and one allele from *T. angustifolia* at all six loci were classed as F_1_ hybrids (*T. × glauca*). Ten plants had other combinations of alleles and were deemed to be either backcrosses to *T. latifolia* or *T. angustifolia* or advanced intercrosses (as per Travis et al. 2010 and Kirk et al. 2011); these plants were omitted from the statistical tests involving species-level differences presented below.

### Statistical analysis

To support our species assignments, a principal components analysis that incorporated all six microsatellite loci was carried out using GenAlEx v. 6.41 (Peakall and Smouse 2006). The first principal component explained 67% of the variation, and the parental species were well separated according to the first axis (Appendix 1). Individuals classified as *Typha latifolia* had PC1 scores <–0.4, while those classified as *T. angustifolia* had PC1 scores >0.4. Clustering of parental species therefore agreed with species’ assignments based on the private alleles method described by Kirk et al. 2011). Plants with intermediate PC1 scores always possessed private alleles from both parental species; these were classified as hybrid *T. × glauca*.

To enable comparisons of elevations across sites with different absolute elevations, our estimates were standardized by subtracting the median shoot elevation score per site from each sample measurement. We evaluated whether *T. latifolia* occurred at different elevations than *T. angustifolia* and *T. × glauca* using a linear mixed model. The model was calculated using the lme function in the nlme library (Pinheiro et al. 2011) in R (R Development Core Team 2011). Elevation was the dependent variable, species was the fixed independent variable, and shoot height was included as a covariate. Site and genet (nested within site) were included as random grouping variables. We assumed that multilocus genotypes from the same population differing by only one allele were members of the same genet, a method that has been shown to effectively compensate for genotyping errors in clonal plants (Schnittler and Euseman 2010). This reduced the total number of inferred genets by nine (i.e., to 86 from 95). We evaluated differences in genotypic diversity between species using a generalized linear mixed model (GLMM). The model was calculated using the lmer function in the lme4 library (Bates et al. 2011) with the ratio of identified genets to the total number of ramets sampled as the dependent variable, and species as the independent variable. We specified a binomial error term (and a logit link function) and included site as a random grouping variable. The results of this test are reported in terms of the genet richness (*R*) for each species *i* per site *j*:

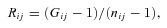
1
where *G* is the number of multilocus genotypes detected, and *n* is the sample size. The value of *R* varies from 0 when all *n* ramets in a sample possess the same genotype, to 1.0, when all ramets possess a different genotype (Dorken and Eckert 2001). Finally, we evaluated whether the distribution of elevations occupied by *T. latifolia* ramets was shifted towards shallower elevations in comparison with *T. angustifolia* using a Kolmogorov–Smirnov test, calculated using the ks.test function in R.

## Results

**Figure 2:**
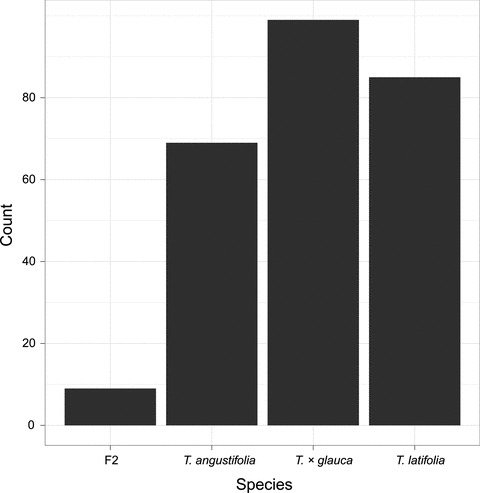
Frequencies of *Typha* spp. encountered across the 18 sites in this study. All later generation hybrids and apparent parental backcrosses are grouped together as F2s.

The most common plants in our sample were hybrid *T. × glauca*, followed by *T. latifolia* and *T. angustifolia* (Fig. 2). Only a small proportion of shoots were apparent backcrosses or advanced intercross hybrids (9 of 262 shoots), which were omitted. Although our estimate of genet richness for *T. latifolia* was nearly 2× higher than the other species (*R*= 0.27 ± 0.07 SE for *T. latifolia* vs. *R*= 0.14 ± 0.04 SE for *T. × glauca* and *R =* 0.15 ± 0.06 SE for *T. angustifolia*), the GLMM failed to detect a significant difference between species (*T. angustifolia* vs. *T. × glauca*: parameter estimate =–0.07 ± 0.33 SE, Wald's *Z*=–0.22, *P*= 0.82; *T. angustifolia* vs. *T. latifolia*: parameter estimate = 0.33 ± 0.31 SE, Wald's *Z*= 1.04, *P*= 0.30).

**Figure 3:**
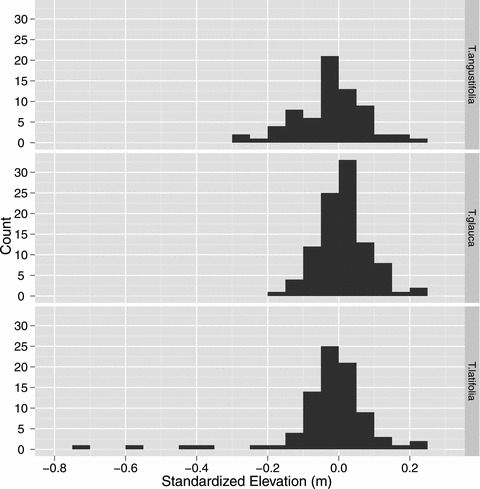
Distributions of standardized shoot elevations for *Typha angustifolia*, *T. × glauca*, and *T. latifolia* measured across the 18 sites included in this study. Shoot elevations were standardized by subtracting the median shoot elevation score per site from each sample measurement.

We found no evidence to support the niche segregation hypothesis for the maintenance of *T. latifolia*. We detected broad overlap in shoot elevations among the three species within sites (Fig. 3) and there was no significant difference in shoot elevations among species (linear mixed-effects model: *F*_2,166_= 2.46, *P*= 0.09). Moreover, there was no evidence from the Kolmogorov–Smirnov tests for a difference in the distribution of shoot elevations between *T. latifolia* and *T. angustifolia* (*D*= 0.12, *P* > 0.50) or between *T. latifolia* and *T. × glauca* (*D*= 0.18, *P >* 0.05). Indeed, on average, *T. latifolia* was observed to occupy elevations remarkably similar to those occupied by *T. angustifolia* (average standardized elevation for *T. latifolia*=–2.6 cm vs. –2.0 cm for *T. angustifolia*)*,* while *T. × glauca* had the highest average standardized elevation (+1.2 cm). Finally, there no association between plant height and standardized elevations within sites (linear mixed-effects model: *F*_1,166_= 3.03, *P* > 0.05).

## Discussion

Niche segregation of closely related species in heterogeneous environments may provide a mechanism for the maintenance of species boundaries, and may promote the maintenance of species diversity (Chesson 2000). In this study, we found no evidence for niche segregation among cattail species and their hybrids across elevational gradients within sites, and therefore no evidence to support the niche segregation hypothesis for *T. latifolia*. Moreover, our data failed to provide any support for the frequently reported (but rarely measured) difference in shoot elevations between *T. latifolia* and *T. angustifolia*. Our data, from 18 sites, show that the average shoot elevations for each species are remarkably similar, particularly for *T. latifolia* and *T. angustifolia*. Below, we discuss these findings in more detail and consider their implications for understanding the dynamics of *Typha* hybrid zones.

### Water-depth gradients and cattails

Although differences in water-depth preferences between cattail species are widely reported, there are only limited data to support these claims. The most detailed comparison of the distribution of cattail species across a water-depth gradient was conducted by Grace and Wetzel (1981), who evaluated the occurrence of *T. latifolia* and *T. angustifolia* at five intervals between –15 cm (i.e., above water) and 100 cm below the water surface in an experimental pond. They showed a clear predominance of *T. latifolia* in shallower water and of *T. angustifolia* in deep water and, using transplanted ramets, that these contrasting water depths provided better growth conditions for each species. Travis et al. (2010) have similarly demonstrated a difference in the relative abundance of *T. latifolia* versus other cattails across a water-depth gradient in a natural stand, with *T. latifolia* occurring more frequently in shallower water and other cattails more frequently in deep water. In both cases, inferences about species differences came from a single site. Although our data come only from a limited number of samples per site, our measurements from 18 sites revealed remarkably similar shoot elevations among species.

A further complicating factor for previous reports of differences between species in water-depth preferences involves the use of phenotypic markers to infer species identities. With the exception of the study by Travis et al. (2010), previous inferences regarding the water-depth preferences of cattails have the potential to misrepresent actual patterns because of the exclusive use of phenotypic characters to identify species. Hybrid cattails can appear remarkably similar to either parental species, and species identification using only gross morphological characters is often inconclusive (Kuehn et al. 1999; Olson et al. 2009). Accordingly, in the absence of molecular genetic data to corroborate species designations, anecdotal or even experimental reports of differences in the performance of cattails across water-depth gradients might be of limited utility.

A potential limitation of our study was that we did not measure water depths directly. However, this would have been impossible given the nature of the sampling conducted. We attempted to target mixed cattail stands for sampling and, because we used phenotypic characters to provide preliminary species assessments, were therefore limited to sampling sites post flowering. By this time, water levels in many of the wetlands in the study region have already begun to drop. As a result of these seasonal fluctuations, single point measurements of water depths complicate comparisons across sites, where, in some cases, there may be no standing water at the time of sampling (note that in the spring, and prior to the seasonal drying of the wetlands in this region, all of our sites were covered by standing water; A. McKenzie-Gopsill, personal observation). As already noted, because elevations were measured only for cattails occurring in contiguous habitat, elevations should directly correspond with water depths before water levels begin to fall later in the summer.

### Hybridization and the evolution of invasive cattails

The importance of niche segregation for the maintenance of reproductive isolation between closely related species, despite opportunities for interspecific gene flow, is widely recognized (Anderson 1948; Funk et al. 2006; Mallet 2008). Several notable examples where congeneric species and hybrids are spatially segregated according to ecological variables include Louisana irises (Anderson 1948; Cruzan and Arnold 1993) and sunflowers (Rieseberg et al. 2003). However, data from this study, combined with the observation that mixed stands of cattails in our study region are common, suggest that if there is any ecological differentiation between *T. latifolia, T. angustifolia,* and their hybrids, it is weak. Moreover, many wetlands in the region surrounding the Great Lakes are subject to anthropogenic disturbance, often in the form of nutrient inputs from agriculture and alterations of drainage patterns for development. These changes have been associated with substantial increases in the abundance of emergent aquatic macrophytes, including *Lythrum salicaria*, *Phragmites australis* (Trebitz and Taylor 2007), and cattails (Woo and Zedler 2002; Craft et al. 2007). Such anthropogenic disturbances are thought to promote the breakdown of ecologically based barriers between species and promote the occurrence of hybridization (Anderson 1948; Taylor et al. 2006; Gilman and Behm 2011). The relative fitness of *T. × glauca* compared to its parental species has not been directly measured across a range of water depths, however a high abundance of hybrids reported in this and other studies (Travis et al. 2010; Kirk et al. 2011) suggests that *T. × glauca* is not less fit overall compared to parental species in most habitats where it is found. Indeed, increases in the abundance of cattails over the past century are also associated with increases in the abundance of hybrid *T. × glauca* (Boers and Zedler 2008, and references therein).

The occupation of divergent niches by hybrids and their progenitor species often results in the occupation of different (but perhaps overlapping) geographic ranges (e.g., Rieseberg 2006). However, if species occupy similar niches, why should they not then occupy similar ranges? Studies by Travis et al. (2010) and Kirk et al. (2011) have both suggested that *T. × glauca* is more abundant than either parental species in regions close to the Great Lakes of North America. By contrast, *T. latifolia* is more prevalent than either *T. angustifolia* or *T. × glauca* in regions closer to the Atlantic coast (e.g., New Brunswick, Nova Scotia, and Maine; Kirk et al. 2011). The introduced species *P. australis* occupies similar habitats as cattails and does particularly well in nutrient-enriched environments, possibly because of its historic distribution in eutrophic Eurasian wetlands (Holdredge et al. 2010; Mozdzer and Zieman 2010). Growing evidence suggests a similar trend in *Typha* (Boers and Zedler 2008, and references therein). This may reflect historical patterns of isolation, combined with ongoing redistribution of lineages via anthropogenic and natural means. Alternatively, niche segregation may occur according to one or more environmental variables that were not measured in this study. For example, other studies have suggested correlations between the success of *T. × glauca* and elevated soil organic matter, soil nutrients, and the presence of beach ridges (Lishawa et al. 2010), factors that were not considered in our study. Also, this study measured only the distribution of each lineage at sites where all three lineages are sympatric, and the hybridization process is presumably ongoing. The distribution of lineages within such sites might be influenced by stochastic demographic processes including random patterns of seed dispersal, and we may not have captured the long-term effects of selection resulting from competition between the lineages, particularly if such selection pressures are weak.

The widespread hybridization revealed in this species complex by Travis et al. (2010, 2011) and Kirk et al. (2011) appears to be a relatively recent phenomenon in North America (Shih and Finkelstein 2008). It is not yet clear why hybridization events might have become more frequent, although anthropogenic land use changes have been implicated (Farrell et al. 2010). In addition, although *T. angustifolia* is widely reported to flower earlier than *T. latifolia* (Grace and Harrison 1986; Selbo and Snow 2004), some sites in the Great Lakes region in which hybridization has been identified are characterized by highly synchronous flowering times between the two parental species (D. Ball and J. R. Freeland, unpublished data). In this study and in previous studies (Travis et al. 2010; Kirk et al. 2011), advanced generation backcrosses and intercrosses are present but are uncommon relative to F_1_ hybrids. Nonetheless, even low levels of backcrossing can facilitate the introgression of genomic regions between species (Rieseberg et al. 2007). Taken together, the apparently recent advent of widespread hybridization and the apparent lack of barriers to interbreeding that could occur through niche segregation suggest that cattails may provide a useful example of reverse speciation (as per Seehausen et al. 2008), and one with implications for understanding the spread of invasive plant lineages.

## Appendix 1

Principal components analysis clearly separates the two parental species and their F_1_ hybrid. Plants classified as *Typha latifolia* had PC1 scores less than −0.4, and those classified as *T. angustifolia* had PC1 scores greater than 0.4. Hybrids had intermediate PC1 scores and are clustered toward the center of the diagram. The intensity of the shading of each dot corresponds with the number of individuals with the same PC1 and PC2 scores; these individuals represent plants that were not distinguishable with the six microsatellite loci used in this study.
